# Hypothesis: NDL proteins function in stress responses by regulating microtubule organization

**DOI:** 10.3389/fpls.2015.00947

**Published:** 2015-10-31

**Authors:** Nisha Khatri, Yashwanti Mudgil

**Affiliations:** Plant Molecular Biology Lab, Department of Botany, University of DelhiNew Delhi, India

**Keywords:** N-MYC DOWNREGULATED GENE, N-MYC DOWNREGULATED-LIKE, phospholipase D, phosphatidic acid, microtubule assembly, microtubule-associated protein, abiotic stress

## Abstract

N-MYC DOWNREGULATED-LIKE proteins (NDL), members of the alpha/beta hydrolase superfamily were recently rediscovered as interactors of G-protein signaling in *Arabidopsis thaliana*. Although the precise molecular function of NDL proteins is still elusive, in animals these proteins play protective role in hypoxia and expression is induced by hypoxia and nickel, indicating role in stress. Homology of NDL1 with animal counterpart N-MYC DOWNREGULATED GENE (NDRG) suggests similar functions in animals and plants. It is well established that stress responses leads to the microtubule depolymerization and reorganization which is crucial for stress tolerance. NDRG is a microtubule-associated protein which mediates the microtubule organization in animals by causing acetylation and increases the stability of α-tubulin. As NDL1 is highly homologous to NDRG, involvement of NDL1 in the microtubule organization during plant stress can also be expected. Discovery of interaction of NDL with protein kinesin light chain- related 1, enodomembrane family protein 70, syntaxin-23, tubulin alpha-2 chain, as a part of G protein interactome initiative encourages us to postulate microtubule stabilizing functions for NDL family in plants. Our search for NDL interactors in G protein interactome also predicts the role of NDL proteins in abiotic stress tolerance management. Based on published report in animals and predicted interacting partners for NDL in G protein interactome lead us to hypothesize involvement of NDL in the microtubule organization during abiotic stress management in plants.

## Introduction

An average estimated yield loss by abiotic stress is more than 50% across the world, caused mainly by salinity, drought and temperatures (Boyer, 1982). Matter of concern is that global population is likely to reach 10 billion by 2050 (almost doubled) (Tilman et al., 2002). So the generation of stress tolerant plants is the need of the hour (Smedema et al., 2000). Salinity is the most destructive and complex stress, affects more than 45 million hectares of irrigated land worldwide, in INDIA about 8.6 million hectare area is affected by salinity (Pathak, 2000).

Right from the beginning of seed germination till crop yield, salt stress affects plant adversely via ionic imbalance leading to toxicity, nutritional disorder, hampering metabolic processes, osmotic stress leading to membrane disorganization, reduction of cell divisionand expansion, and oxidative stress (Hasegawa et al., 2000; Duan et al., 2015; Khare et al., 2015).

**FIGURE 1:**
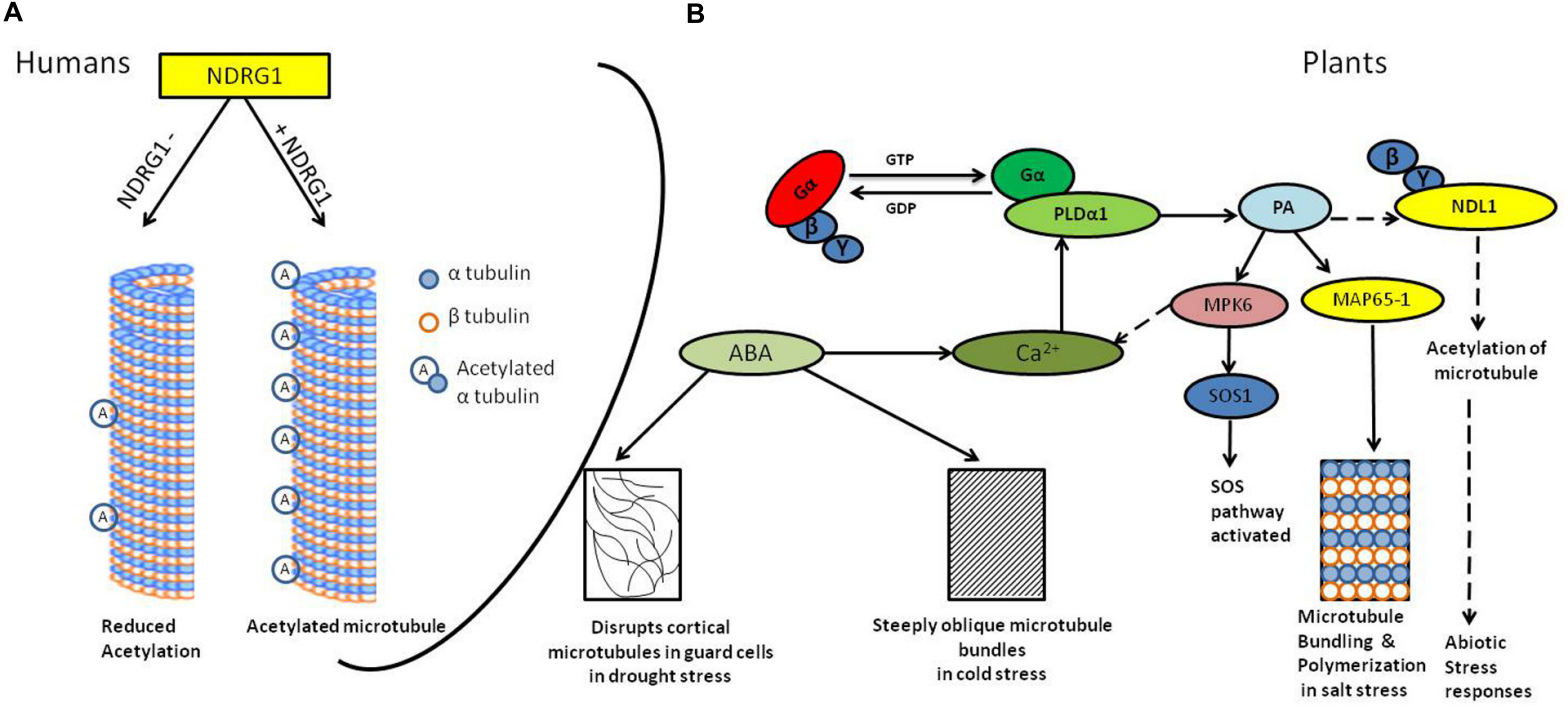
**Diagrammatic representation of the signaling in abiotic stress and microtubule responses.** **(A)** N-MYC DOWNREGULATED GENE (NDRG1) knock-down human mammary epithelial cells (hNMECs) shows decrease in tubulin acetylation as compared to cells having wild type levels of NDRG1(Kim et al., 2004). **(B)** Abiotic stress activates G-protein-coupled receptors (GPCRs; Yadav and Tuteja, 2011), *phospholipase-D*α1 (PLDα1) interacts with activated Gα subunit (Zhao and Wang, 2004); PLD hydrolyzes membrane lipids to generate phosphatidic acid (PA). PA binds to MAP65-1, resulting into microtubule bundling and polymerization which helps in salt tolerance (Zhang et al., 2012) it also activates MPK6, which further phosphorylates SOS1 resulting into activation of SOS pathway (Yu et al., 2010). PA may interact with NDL1, interactor of Gβγ dimer and possible downstream regulator of microtubules. ABA production during cold and drought stress results into steeply oblique and disrupted microtubules, respectively, (Wang and Nick, 2001; Pollock and Pickett-Heaps, 2005). Solid lines depicts confirmed interactions, dotted line depicts hypothesized interactions

Although, the role of lipids in salt stress is not well understood, it has been indicated that expression of several *phospholipase-D* (*PLD*) genes is induced by salt stress (Katagiri et al., 2001; Hong et al., 2010). Hydrolysis product of PLD, phosphatidic acid (PA) is shown to bind and activate mitogen-activated protein kinase 6 (MPK6), which in turn phosphorylates salt overly sensitive 1 (SOS1) transporter *in vitro* (**Figure 1**; Yu et al., 2010). The SOS1 gene encodes a plasma membrane Na+/H+ antiporter, playing protective role in saline environment. These findings have indicated a link between lipid signaling, MAPK cascades, and salt stress tolerance in plants (Morris, 2010). Plant responses to salt stress include osmolyte biosynthesis, water flux control, and transport of ions for re-establishment of homeostasis and microtubule depolymerization and reorganization (Wang and Nick, 2001; Lü et al., 2007; Wang et al., 2007, 2010). Although all of the events are equally important for cell survival, microtubule depolymerization and reorganization are believed to be essential for plant survival under abiotic stress.

## Ndrg As A Microtubule-Associated Protein (Map)

Microtubule organization is regulated by MAPs (Dixit and Cyr, 2004; Sedbrook, 2004). In animals, several MAPs have been identified and characterized. Detailed analysis of human N-MYC DOWNREGULATED GENE (*NDRG*) gene family showed that the family comprises of four members (*NDRG1-4*), each sharing 57–60% amino acid sequence similarity (Qu et al., 2002). Among these, only NDRG1 has been reported to be a MAP which participates in the spindle checkpoint in animals (Kim et al., 2004).

Microtubule dynamics is affected by an array of reversible post-translational modifications including acetylation, phosphorylation, and palmitoylation (Piperno et al., 1987; Westermann and Weber, 2003; Zhang et al., 2003). Acetylated tubulin is one of the major characteristics of stabilized microtubule structure and may contribute to regulating microtubule dynamics (Westermann and Weber, 2003; Parrotta et al., 2014). Mammalian NDRG1 knockdown cell line have decreased accumulation of acetylated -tubulin and disrupted spindle fiber formation (**Figure 1**; Kim et al., 2004). Moreover, growing body of evidences also show that NDRG1 recruits on recycling endosomes in the Trans Golgi Network by binding to phosphatidylinositol 4-phosphate and interacts with membrane bound Rab4aGTPase (Kachhap et al., 2007). Kachhap et al. (2007) used a prostate cancer cell line to show that NDRG1 is a novel effector for the small GTPase, Rab4a, and is important in recycling E-cadherin in proliferating cells.

## Structural Similarities Between Ndrg1 And Ndl1

In plants, NDL proteins were first reported in sunflower (SF21) as stigma and transmitting tissue cell specific proteins (Kräuter-Canham et al., 1997). Thereafter, studies on SF21 proteins identified it as a small gene family with putative role as a signaling molecules in pollen-pistil interaction. Across plant species, *SF21* gene has been reported in dicots (*Lycopersicon esculentum*, *Arabidopsis thaliana*) monocots (*Oryza sativa*) (Lazarescu et al., 2006), gymnosperms as well as in the moss, *physcomitrella patens* (Lazarescu et al., 2010). *Arabidopsis NDL* gene family has three members *NDL1, NDL2*, and *NDL3*. All family members contain NDR domain, an alpha/beta hydrolase fold, a conserved hydrophobic patch of 23 amino acids and a conserved Asp. All these mentioned features strongly suggest that NDL proteins belong to NDR protein family. NDL proteins in *A. thaliana* are novel effectors of G-protein signaling playing important role in root and shoot development (Mudgil et al., 2009, 2013). G-protein core complex relay signal intracellularly with the help of downstream effectors or secondary messengers.

We previously observed that Mouse NDRG1 interacts with *Arabidopsis* AGB1/AGG1 and AGB1/AGG2, suggesting that this interaction is evolutionarily conserved (Mudgil et al., 2009). Human NDRG1 is 93% similar to mouse NDRG1 (Mudgil et al., 2009), so we can postulate similar interaction of human NDRG1 with plant’s G protein components. Also, NDL in *Arabidopsis* and NDRG1 of mouse were shown to interact with the C-terminal domain of regulator of G-protein signaling (RGS1), a candidate seven-transmembrane receptor in AGB1/NDL-mediated signaling via yeast two-hybrid (Mudgil et al., 2009).

N-MYC DOWNREGULATED GENE1 functions as a MAP and acetylates microtubules in human. NDRG1 also act as novel effector for the small GTPase. In plants, protein domains search revealed that all α tubulin family subunits contain GTPase domain as the tubulin C terminal domain so NDL might also interact with α tubulin in plants.

## Microtubules Dynamics-Role In Abiotic Stress Tolerance

Microtubules are the polymers of heterodimeric protein αβ-tubulin, which provides shape to cells and maintains tracks for vesicle transport and segregation of chromosome. Microtubule organization is regulated by microtubule-associated proteins (MAPs; Dixit and Cyr, 2004; Sedbrook, 2004). A variety of MAPs have been reported in higher plants. The MAP65 family and some of kinesin family are important in bundling and polymerization of the microtubules (Smertenko et al., 2004; Van Damme et al., 2004; Mao et al., 2005; Hamada, 2007) *A. thaliana* genome contains nine *MAP65*-related genes with different functions (Hussey et al., 2002).

Calcium is a well-known second messenger which participates in the stress signaling in plants (Knight, 2000; Xiong et al., 2002; Chinnusamy et al., 2005). Cortical microtubules have been suggested to regulate the calcium levels in the cells by regulating the activity of calcium channels (Thion et al., 1996; Himschoot et al., 2015). Treatment of microtubule-destabilizing drug improved the survival and growth of *A. thaliana* seedlings under salt stress while treatments with microtubule-stabilizing drug caused salt stress hypersensitivity (Wang et al., 2007). Moreover, reorientation of microtubules was also observed in maize roots and tobacco BY-2 cells upon short term exposure to salt stress (Blancaflor and Hasenstein, 1995; Dhonukshe et al., 2003). In *A. thaliana*, long term salt stress affected the cortical microtubule organization. *spr1* mutant, [*SPIRAL1*(*SPR1*), a plant-specific MT-localizing protein] has right-handed helical root growth phenotype, salt stress suppresses this phenotype (Shoji et al., 2006). Directional cell expansion (anisotropic growth) is necessary for plant morphogenesis which is achieved by well-organized interphase, cortical microtubule and SPR1 is thought to control anisotropic cell expansion through MT arrangements (Nakajima et al., 2004, 2006). Mutation in critical amino acids of tubulin gene family (mainly located at longitudinal interface of the α and β tubulins), in lateral contact region and in GTPase-activating region in α tubulin (Ishida et al., 2007) disrupts the proper organization and hence functions of microtubules (Hashimoto, 2013). Tubulin mutations affect cortical microtubule arrays in interphase resulting into altered directional growth. Mutation in TUA genes, α tubulin 6 and α tubulin 4 results into right handed helical array of cortical microtubules producing left handed helical growth phenotype, lefty 1 and lefty 2, semi dominant skewing mutants (Thitamadee et al., 2002). These results indicated that the proper organization of microtubule is one of the critical factors for growth and development.

In addition, abscisic acid (ABA), which is produced in response to salt stress, also affects the organization of cortical microtubules (Sakiyama and Shibaoka, 1990; Shibaoka, 1994). In drought stress accumulation of ABA is one of the most pronounced ways to cope up with water deficit stress. ABA leads to stomata closure thereby decrease the water loss and also enhances water uptake by root (Boudsocq and Laurière, 2005). Dehydration triggers plasmolysis of cells and it consequently destroys microtubule (Pollock and Pickett-Heaps, 2005), ABA also disrupts cortical microtubules in guard cells, but not in epidermal cells (Jiang et al., 1996). During cold stress in wheat (Chinese winter wheat) ABA produced steeply oblique microtubule bundles (**Figure 1**; Wang and Nick, 2001).

Phospholipase D is involved in the rearrangement of cortical microtubules (Dhonukshe et al., 2003). In *A. thaliana pldα1* salt-sensitive mutant cortical microtubule showed massive depolymerization patterns (Bargmann et al., 2009; Yu et al., 2010) compared to wild type control. However, upon salt removal from the growth medium organization was recovered in wild-type plants but not in *pldα1* plants indicating involvement of PLDα1 in reorganizing microtubules after depolymerization induced by salt stress (Zhang et al., 2012).

Phosphatidic acid, the end product of PLDα reaction, is a key regulator of microtubule polymerization; exogenous application of PA lead to recovery in salt-disrupted microtubule arrays in *pldα1* mutant (Zhang et al., 2012). PA regulates microtubule bundling and polymerization together with MAP65-1 and their interaction is important for salt tolerance. PA could not bind or bundle microtubules and rescue microtubule disruption caused by salt in the *map65-1* mutant, suggesting that MAP65-1 is necessary for PA-mediated stabilization of microtubules (Zhang et al., 2012). There are two contradictory reports regarding interaction of tubulin and PA. In the first report, a mass spectrometry based approach was used to identify the PA binding proteins which showed that TUA2 is PA binding protein (Testerink et al., 2004). However, in the second report, it was found that neither PLDα1 nor PA species bound to either α- nor β- tubulins. MAP65-1, a microtubule associated protein, was shown to bind to PA but not to other phospholipids like diacylglycerol, phosphatidylserine, phosphatidylinositol, phosphatidylethanolamine, or Phosphatidylcholines. These results indicate that PA requires other MAP to interact with microtubules (Zhang et al., 2012), further experimentation to confirm involvement/role of other MAPs is awaited.

Our analysis of existing information on NDL1 interactome shows interaction with Annexin 1 (ANNAT1) which has role in drought stress (Konopka-Postupolska et al., 2009), sodium and lithium-tolerant 1 (SLT1) which is involved in salt stress (Matsumoto et al., 2001) whereas lesion stimulating disease 1(LSD1) regulates cell death trigged by cold stress (Huang et al., 2010), O-Acetylserine (THIOL) Lyase (OAS-TL) Isoform A1 (OASA1) shows increased cadmium tolerance (Domínguez-Solís et al., 2001) and *Arabidopsis* Ribosomal Protein S27 (ARS27A) is involved in genotoxic stress (Revenkova et al., 1999). Also, comparative analysis shows overlap of NDRG1 and NDL1 interactors involved in similar pathways (**Table 1**).

**Table 1:** N-MYC DOWNREGULATED GENE (NDRG1) and N-MYC DOWNREGULATED-LIKE (NDL1) shared interactors which are involved in common pathways/processes.

	NDRG1 a	NDL1 b	Reference


Cyclin-dependent kinases	Cyclin-dependent kinase 15	Cyclin-dependent kinase – G1 Cyclin-dependent kinase regulatory subunit 2	**a** (Huttlin et al., 2015)
			**b** (Klopﬄeisch et al., 2011)
Calcium-dependent phospholipid binding proteins	Annexin A5	Annexin 1	**a** (Havugimana et al., 2012)
			**b** (Klopﬄeisch et al., 2011)
Heat shock protein	HSPA4 HSPA5 HSP90AA1	BOBBER 1	**a** (Tu et al., 2007; Ambrosini et al., 2009)
			**b** (Klopﬄeisch et al., 2011)
Eukaryotic translation initiation factor	Eukaryotic translation initiation factor 2	Eukaryotic initiation factor 4A-III	**a** (Tu et al., 2007; Kristensen et al., 2012)
	Eukaryotic translation initiation factor 3	DEAD-box ATP-dependent RNA helicase 2	**b** (Klopﬄeisch et al., 2011)
	Eukaryotic translation initiation factor 4H		
	DEAD (Asp-Glu-Ala-Asp) box helicase 1		
	DEAD (Asp-Glu-Ala-Asp) box helicase 5		
	DEAD (Asp-Glu-Ala-Asp) box polypeptide 39B		
Protein phosphatases	Protein phosphatase 2, regulatory subunit B, alpha	protein phosphatase 2A subunit A2	**a** (Tu et al., 2007)
			**b** (Klopﬄeisch et al., 2011)
Components of cytoskeleton machinery	ACTG1, Actin, gamma 1 kinesin family member 5B	TUA2, Tubulin alpha-2 chain KINESIN LIGHT CHAIN-RELATED 1	**a** (Tu et al., 2007)
			**b** (Klopﬄeisch et al., 2011)
Glutathione reductases	Glutathione reductase HEL-75	HOT5, S-nitrosoglutathione reductase	**a** (Kristensen et al., 2012)
			**b** (Klopﬄeisch et al., 2011)
Fatty acid pathway	Fatty acid synthase (FASN)	KCS9 (3-KETOACYL-COA SYNTHASE 9); acyltransferase/catalytic/transferase, transferring acyl groups other than amino-acyl groups	**a** (Tu et al., 2007; Kristensen et al., 2012)
	Acyl-CoA synthetase long-chain family member 3 (ACSL-3)		**b** (Klopﬄeisch et al., 2011)
	Acyl-CoA thioesterase 7 (ACOT7)	Lipoxygenase (LOX2)	
Salinity response	ATPase, Na+/K+ transporting, alpha 1 polypeptide	SLT1 (sodium- and lithium-tolerant 1)	**a** (Tu et al., 2007)
			**b** (Klopﬄeisch et al., 2011)


Our proposed hypothesis that NDL might be playing role in stress mediated processes by regulating microtubule organization (**Figure 1**) can be easily tested by checking NDL1 effect on microtubules bundling and polymerization *in vitro* using purified NDL1 and tubulin proteins. Already available *ndl* loss of function mutants can be used for checking and comparing status of acetylated tubulin in the absence and presence of *NDL*. Effects of various stress responses on tubulin pattern in relation to *NDL* levels can be further studied by analyzing GFP-tagged α tubulin (35S: GFP-TUA2) patterns in *NDL* up and downregulated backgrounds.

## Conflict of Interest Statement

The Guest Associate Editor Girdhar Kumar Pandey declares that, despite being affiliated with the same institute as the authors Nisha Khatri and Yashwanti Mudgil, the review process was handled objectively. The authors declare that the research was conducted in the absence of any commercial or financial relationships that could be construed as a potential conflict of interest.
